# Extraction of Natural Dye from Aerial Parts of Argy Wormwood Based on Optimized Taguchi Approach and Functional Finishing of Cotton Fabric

**DOI:** 10.3390/ma14195850

**Published:** 2021-10-06

**Authors:** Faizan Shafiq, Amna Siddique, Md. Nahid Pervez, Mohammad Mahbubul Hassan, Vincenzo Naddeo, Yingjie Cai, Aiqin Hou, Kongliang Xie, Muhammad Qamar Khan, Ick-Soo Kim

**Affiliations:** 1 College of Chemistry, Chemical Engineering and Biotechnology, Donghua University, Shanghai 201620, China; faizan@mail.dhu.edu.cn; 2 School of Engineering and Technology, National Textile University, Faisalabad 38000, Pakistan; amnasiddique104@hotmail.com; 3 Sanitary Environmental Engineering Division (SEED), Department of Civil Engineering, University of Salerno, via Giovanni Paolo II 132, 84084 Fisciano (SA), Italy; perveznahidmd@gmail.com (M.N.P.); vnaddeo@unisa.it (V.N.); 4 Bioproduct and Fiber Technology Team, AgResearch Limited, 1365 Springs Road, Lincoln, Christchurch 7647, New Zealand; mahbubul.hassan@agresearch.co.nz; 5 Hubei Provincial Engineering Laboratory for Clean Production and High Value Utilization of Bio-Based Textile Materials, Wuhan Textile University, Wuhan 430200, China; yingjiecai@wtu.edu.cn; 6 National Engineering Research Center for Dyeing and Finishing of Textiles, Donghua University, Shanghai 201620, China; aiqinhou@dhu.edu.cn; 7 Nanotechnology Research Group, Department of Textile and Clothing, Faculty of Engineering and Technology, National Textile University Karachi Campus, Industrial Area Korangi, Karachi 74900, Pakistan; 8 Division of Frontier Fiber, Institute of Fiber Engineering, Interdisciplinary Cluster for Cutting Edge Research (ICCER), Faculty of Textile Sciences, Shinshu University, Tokida 3 15 1, Ueda, Nagano 386 8567, Japan

**Keywords:** argy worm wood extracts, natural dye, cotton fabric, taguchi method

## Abstract

The aerial parts of the Argy Worm Wood (AWW) plant have been used in different Chinese foods as a colorant and a taste enhancer for a long time. Despite its application as a food colorant, it has rarely been considered for the coloration of textiles. Keeping in mind the variation in color strength due to the change in phytochemical contents by seasonal change and other variables, the extraction of AWW aerial parts was optimized using the Taguchi method. Optimization was performed on the basis of total phytochemical contents (phenols, flavonoids, and tannins) in the extracted solutions. For this purpose, two different solvent systems, namely sodium hydroxide/water (NaOH/water) and ethanol/water (EtOH/water), were applied through a simple aqueous extraction method at varying levels of solvent concentration, and extraction temperature and duration. Maximum phytochemicals yield of 21.96% was obtained using NaOH/water system with 9 g/L NaOH/water at 85 °C for 20 min and 25.5% with 75% aqueous ethanol at 85 °C for 40 min. Optimized extracts were characterized by UV-Vis and FTIR spectrophotometry, which showed the presence of multiple phytochemicals in the extracts. The dyeing temperature and time were also optimized. Dyed cotton fabrics showed medium to high colorfastness to washing and excellent antibacterial and UV radiation absorption properties. The effect of pre-mordanting with salts of iron and copper was also studied on the color fastness properties. Cotton fabrics dyed with two different solvent system extracts displayed various shades of brown with NaOH/water, and green with aqueous ethanol with and without pre-mordanting. The present study provides the textile industry with a promising source of functional bio-colorant and a value-adding approach for the AWW plant industry.

## 1. Introduction

Clothing is one of the basic needs of a human being. It started from covering the body with tree leaves, but this necessity has turned into fashion over time. With time, people change their clothes according to the trend of the day. People nowadays love to have a full range of colors in their wardrobe. This approach has created a significant demand for the production of colored fabrics using various types of synthetic dyes because of their wide ranges of color, excellent colorfastness properties, convenience, and color reproducibility [1,2,3]. Consequently, a huge amount of hazardous colored effluent is produced by the textile dyeing industry, which has been identified as one of the most polluting industries. According to a World Bank report, the textile dyeing industry causes up to 20% of water pollution, as 13–45% of total dyes used in dyeing get released into the environment with the wastewater. This increased pollution causes threats to marine life, which in turn makes human beings vulnerable [4,5,6]. Therefore, the use of less water and hazardous chemicals during the dyeing and processing of textile fabrics is necessary.

The use of natural dye is a viable alternative to synthetic dyes. There are plants, insects, and minerals from which bio-colorants can be extracted and which are environment-friendly and biodegradable. Moreover, many natural dyes have medicinal properties, as they are composed of polyphenols, tannins, flavonoids, etc., which not only impart color to the materials but also exert functional properties including antibacterial, antimicrobial, and antioxidant properties [7,8,9,10]. The main problems for natural dyes to compete with synthetic dyes are low yields, few colors, high cost, and above all, non-reproducibility in color. Moreover, natural dyes have less affinity towards the cellulosic fibers because of similar ionic charges and poor colorfastness to washing and light. To enhance affinity and colorfastness to washing, different mordants are used [11]. Metallic salt such as alum, iron sulphate, and copper sulphate are typical examples of mordanting agents used in dyeing of textiles with natural dyes. In some studies, tannins of gallnut and pomegranate are used as a bio-based mordanting agent [12].

Natural dyes are a mixture of different phytochemicals and their quality and quantity depend on the extraction conditions, such as pH, temperature, time duration of extraction, and the solvent’s nature and concentration [13]. The resulting color in the textile material depends on the composition of phytochemicals in the extracted solution; thus, a change in the process conditions can affect the final color. Little work has been performed on the optimization of extraction and dyeing conditions to reduce the variation in final color in the past. The present study focused on extracting the dye from the aerial parts of Argy wormwood (AWW) (*Artyemisia argyi*), commonly known as Chinese mugwort, a native Chinese medicinal plant with a long history of use in Chinese foods as a colorant and flavor enhancing agent [14,15]. Apart from its usage as a bio-colorant, the crude extract of AWW is used as a therapeutic drug (known as “QI AI”), for the treatment of abdominal pain, menstrual irregularities, asthma, bronchitis, etc. [16]. Previous studies have shown that the main components of AWW leaves are essential oils, flavonoids, and tannins. These components exert antibacterial, antitumor, anti-oxidation, and analgesic properties [17]. A review of the literature shows that the extract of AWW has never been explored systematically as a dye for the coloration of textile fibers.

In the present study, we focused on optimizing the bio-colorant extraction procedure from AWW aerial parts by using two different solvent systems and their application for the dyeing of cotton fabric. There are many synthetic textile materials that have been developed for clothing and apparel, but cotton is still the top choice, because it is comfortable as well as being thermally stable [18,19]. For the optimization process, the Taguchi design of the experiment was carried out using MINITAB statistical software. Optimization was done based on the total phytochemicals present in the extracted solutions. Extracted dyeing solutions were characterized by UV-VIS spectrophotometry. The dyed fabric samples were tested for their color strength (K/S), CIE color coordinates (L*, a*, b*, C* and h°) and color fastness properties according to international standards. Effects of pre-mordanting with two metal mordants (Fe^2+^, Cu^2+^) on the color properties and the functional properties, such as antibacterial and UV protection of dyed cotton fabric, were also tested.

## 2. Materials and Methods

### 2.1. Materials and Chemicals

Dry aerial parts of AWW were purchased from a local market and washed thoroughly with tap water to remove soil and dust. After complete drying in the oven at 50 °C, whole aerial parts were ground into fine powder using a laboratory-scale electric grinder. The powder was kept in an airtight plastic in at a cool place in darkness until further use. Folin–Ciocalteu, gallic acid, and catechin were purchased from Shanghai Macklin Biochemical Co., Ltd. (Shanghai, China). Commercially available 100% pure knitted scoured and bleached cotton fabric (180 GSM) was used for dyeing. Other chemicals such as hydrochloric acid (HCl), sodium hydroxide (NaOH), sodium nitrate (NaNO_2_), ethanol (C_2_H_6_O), aluminum chloride (AlCl_3_.6H_2_O), and copper sulphate (CuSO_4_.5H_2_O) were purchased from China Pharmaceutical Group (Shanghai, China). Ferrous sulphate (FeSO_4_.7H_2_O), sodium carbonate (Na_2_CO_3_), and formaldehyde (CH_2_O) were purchased from Sigma Aldrich (St. Louis, MI, USA).

### 2.2. Extraction and Optimization of Phytochemicals

Preliminary extraction trials were conducted by using four different solvent systems through a simple water bath extraction technique. The color coordinates of the extracts were measured with the help of a Datacolor 650 spectrophotometer (Datacolor International, Rotkreuz ZG, Switzerland). The four different solvent systems and the extraction conditions of temperature and time are given in Table 1. However, solvent concentration, extraction temperature, and time needed to be optimized, for which 5 g powdered raw material was used per 100 mL of solvent for every experiment, and the optimization process was conducted based on the Taguchi design due to its reliability, simplicity and execution ability [20,21]. Extracted solutions were stored in a cool place away from the direct light to avoid unwanted heat reactions.

### 2.3. Phytochemical Analysis

**Figure 1:**
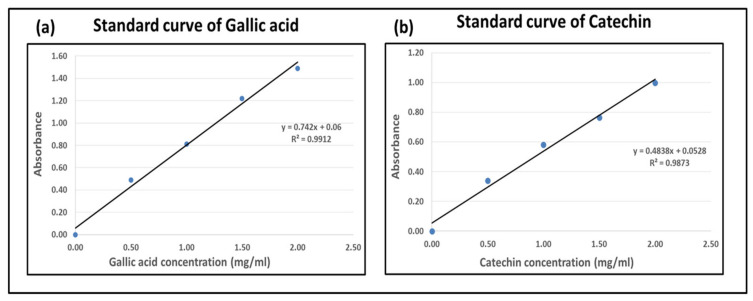
Standard curves: (**a**) gallic acid, (**b**) catechin.

Total phenols and flavones were measured spectrophotometrically using the Folin−Ciocalteu colorimetric method and catechin method, respectively. At the same time, tannins were quantified through the Stiasny reaction. The Folin–Ciocalteu method was adopted as described previously by Xia et al. and Si Tan et al., with modifications [22,23]. Briefly, 1 mL of extracted solution was placed in a test tube. An amount of 0.5 mL of Folin–Ciocalteu reagent was combined with the addition of 10 mL deionized water. After 15 min of continuous stirring, 2 mL of sodium carbonate solution (15%) was added. The mixture was heated to 50 °C for 20 min in a water bath and then cooled down to room temperature. Absorbance values were recorded at a wavelength of 760 nm, and calculations were performed using a regression equation for gallic acid (0.5–2 mg/mL). Results are reported in terms of gallic acid equivalents (GAE). The standard curve of gallic acid is shown in Figure 1a.

Total flavonoid contents in crude plant extracts can be checked using the spectrophotometer [24,25]. Briefly, 1 mL of extract was taken in a test tube. An amount of 1 mL of deionized water was combined with 0.5 mL of Sodium Nitrate (5% solution) in the tube. The mixture was given 15 min for the reaction, and then 0.5 mL of aluminum chloride (10% solution) was added. With continuous stirring, 5 mL of sodium hydroxide (1M solution) was added after 5 min. The mixture was diluted with deionized water, and absorbance was checked at a wavelength of 510 nm. Flavonoids were expressed in catechin equivalents by using the regression equation of catechin standard (0.5–2 mg/mL). The standard curve of catechin is shown in Figure 1b.

Stiasny reaction was carried out for the quantification of total tannins in the extracts. Many researchers have reported the Stiasny reaction [26,27]. Briefly, 50 mL of each extract was taken and put on a magnetic stirrer with 7.5 mL of chloric acid followed by 12.5 mL of Formaldehyde solution. The mixture was stirred for 30 min and then filtered. The residue was put in the oven at 105 °C + 3 °C until a constant mass was obtained. Equation (1) was used to calculate the yield of tannins:

(1) $$\mathrm{SI}\left(\%\right)=\frac{mass\;of\;residue}{\mathrm{initial}\;\mathrm{mass}\;\mathrm{of}\;\mathrm{extract}}\times 100$$

### 2.4. Characterization of the Extracted Dye

Optimized extracts were centrifuged at 10,000 rpm for 10 min. Supernatants were collected and filtered immediately to remove the residues and avoid any unwanted contamination. The extracted solutions from the aerial parts of AWW at optimized extraction conditions were characterized by UV-VIS spectrophotometry. TU-1901 UV−VIS spectrophotometer (Shaanxi, China) was used with wavelengths ranging from 200 to 800 nm. Additionally, the dye was also characterized by Fourier Transform Infrared Spectroscopy with an ATR-FTIR spectrum 2 spectrophotometer (PerkinElmer, MA, USA). Samples were tested in the range of 500–4000 cm^−1^. The background spectra were taken on air before each analysis to minimize the error in the results. Spectra were further analyzed by using Origin Pro software.

### 2.5. Optimization of Dyeing Factors

After the extract optimization, the next step was to determine the optimum conditions for dyeing with the AWW extracts, such as dyeing time and temperature. All the dyeing experiments were carried out with a material-to-liquor ratio of 1:20 at the initial pH of the optimized extracts. To optimize dyeing temperature, 5 samples were dyed at 30, 45, 60, 75, and 90 °C for 60 min each. Furthermore, to optimize the dyeing time, 5 more samples were dyed at the optimized dyeing temperature for different time durations of 40, 60, 80, 100, and 120 min. Dyed fabric samples were washed, soaped, and dried at 60 °C.

Dyeing parameters were optimized based on the color strength (K/S) obtained. The color strength was measured using a reflectance spectrophotometer (Datacolor 650) with the help of the Kubelka–Munk Equation (2), which is as follows:

(2) $$\frac{K}{S}=\frac{{\left(1-R\right)}^{2}}{2R}$$

where ***K*** represents the absorption coefficient, ***S*** represents the scattering coefficient, and ***R*** represents reflectance. Three different readings were taken at various points from the double-folded fabric samples and the average values are reported here.

### 2.6. Mordanting

In our study, we used two different metallic mordants, namely FeSO_4_ and CuSO_4_. Both mordants were applied through pre-mordanting technique at 90 °C for 20 min with 5% mordant on the weight of the fabric sample.

### 2.7. Color Measurement

Dyed fabric samples were subjected to color evaluation by using a Datacolor 650 spectrophotometer. CIE L*, a*, b*, C* and h° coordinates and K/S were measured where L* represents the lightness of the color on a scale of 0 (black) to 100 (white), a* represents the redness (positive) or greenness (negative), b* represents yellowness (positive) or blueness (negative), C and h° stand for chroma and hue of the color, respectively, while K/S is the strength of the color on the fabric.

### 2.8. Colorfastness Properties

Colorfastness to washing, rubbing, and light were evaluated according to ISO Standards. ISO 105-C06 was followed for the wash fastness. Assessment for color change and staining on the adjacent cotton fabric was also done. ISO 105-X12 testing standard was applied to check the rubbing fastness and ISO 105-B02 for the light fastness. Each sample was fitted inside a closed chamber and directly exposed to a Xenon arc lamp for 24 h.

### 2.9. Antibacterial Activity

To quantify the antibacterial activity of the dyed fabric against *E. coli* (Gram-negative) and *S. aureus* (Gram-positive) bacteria, we adopted the measurement of growth of bacteria by measuring the turbidity of the bacterial culture solution [28]. Briefly, the dyed fabric samples were cut in the exact sizes of 1 × 1 cm and UV-sterilized for 3 h before testing. Different tubes were taken and filled with broth culture media. In one tube remained the culture media only and was labeled as a positive control (no bacteria). In contrast, in another tube, bacteria were added and labeled as a negative control. In other tubes, bacteria were added along with the dyed samples and labeled accordingly. All the tubes were then placed in incubation at 37 °C for 24 h while continuously shaking. After 24 h, the optical density was measured for each test tube sample. As optical density and number of bacteria has proportionality between them thus the antibacterial activity was calculated by using Equation (3):

(3) $$\mathrm{Antibactirial activity}\;\left(\%\right)\;=\;\frac{\mathrm{In}-\mathrm{Is}}{\mathrm{In}}\times 100$$

where ***In*** and ***Is*** are the optical densities of the negative control sample and the dyed pieces.

### 2.10. UV Protection Property

To check the UV protection ability of the dyes applied to the cotton fabric, we used the YG912E Textile anti-ultraviolet tester (GOIN International Viet Nam Co., Ltd., Ho Chi Minh City, Vietnam). UPF values of the un-dyed and dyed cotton fabrics were tested according to the EU standard 13758-2001. The transmittance of UV-A and UV-B radiations was recorded from 290–400 nm wavelength with an interval of 10 nm. Five measurements were taken from different surface points on each sample, and an average value was reported.

## 3. Results

### 3.1. Solvent Selection

**Figure 2:**
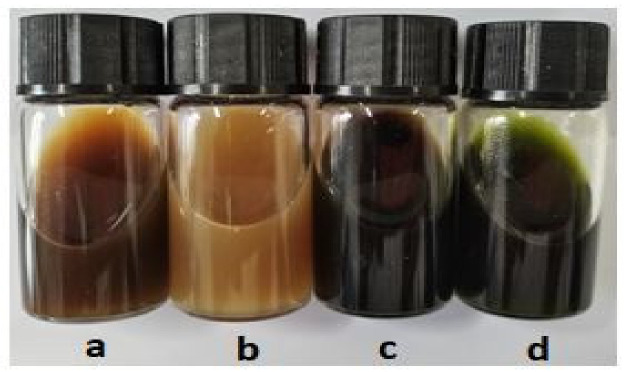
Extracted trial solutions; (**a**) water, (**b**) water/HCl, (**c**) water/NaOH and (**d**) EtOH.

Preliminary extraction trials were performed to compare four different solvent systems according to the extraction conditions given in Table 1. Color coordinates and color strength values of the extracted solutions were measured by using Datacolor 650. Extracted solutions visual representation is shown in Figure 2. 

The color coordinates of the extracted solutions were measured by using Datacolor 650. Results are presented in Table 2.

The results show that water/NaOH and EtOH solvent systems are more efficient as darker colors with high color strengths were achieved. A dark brown color solution with NaOH and a dark green color solution with EtOH were obtained. Plant material consists of different polyphenols (PPs), which can be extracted by using different solvents. The selection of solvent depends on the material from which extraction is to be done. Organic solvents have been widely used for this purpose. Several studies have reported aqueous ethanolic extraction of phytochemicals from different plants [29,30]. In addition to organic solvent extraction, alkaline solvent extraction has been in practice for a long time [31,32]. The current study focused on the dyeing of cotton fabric. As the targeted material is sensitive to acidic pH, NaOH and EtOH solvent systems were used for further optimization. Different percentages of EtOH (0 to 100%) and NaOH (3 to 15 g/L) were applied for the extraction optimization. For NaOH, a concentration higher than 15 g/L was not used as a safety measure to avoid the degradation of the chosen plant material.

### 3.2. Optimization of Extraction Parameters

MINITAB statistical software was used to produce the Taguchi DOE with three extraction parameters and their five levels. Optimization was performed on the basis of the quantity of total polyphenols extracted by different combinations of parameters and their levels, which are presented in Table 3.

A total of 25 experiments were carried out according to the TAGUCHI L25 DOE. The obtained percentage of total polyphenols, i.e., SI% against the applied combination of parameters and their levels, are given in Table 4.

The results show a significant variation in the quantity of total polyphenols obtained depending on the different extraction conditions. In the case of NaOH, the maximum SI% of 21.96% was obtained using 9 g/L NaOH at 85 °C for 20 min, while in the case of EtOH, the top SI% of 25.53% was obtained by using 75% aqueous ethanol at 85 °C for 40 min. MINITAB also helps determine the effect of each parameter on the response individually, which is helpful to measure the contribution of each parameter. Furthermore, by using MINITAB, we can also determine the interaction between the parameters. These tools are helpful for checking the credibility of the obtained results.

### 3.3. Study the Main Effects of Extraction Parameters

**Figure 3:**
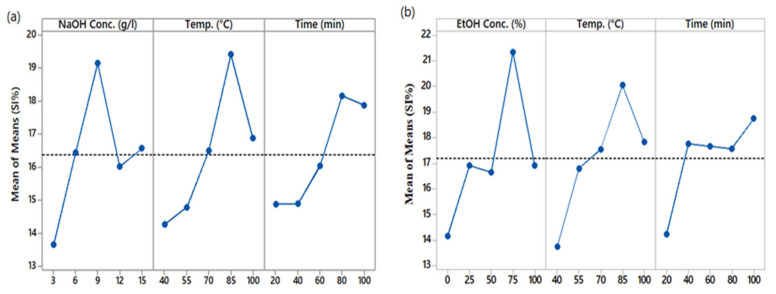
Main effect plots of SI% (**a**) NaOH, (**b**) EtOH.

Figure 3 shows the main effect plot of the extraction parameters for the quantity of polyphenols, represented by SI%, present in the extracted solutions. The inclination of the main plot lines shows the magnitude of the effect of the corresponding parameter on the results. The steeper the line, the higher the magnitude will be, and vice versa. It is clear from Figure 3a that NaOH concentration and temperature are the parameters that had the highest impact on the extraction of polyphenols. On the other hand, extraction time had the most negligible effect. In the case of the ethanol/water solvent system, Figure 3b shows that percentage of ethanol has the highest effect on the response, i.e., SI%. In second place comes the extraction temperature, as it can be seen from the main effect plot of EtOH that the inclination of extraction temperature is less than that of the concentration of EtOH. The most negligible effect is that of extraction time, with the lowest inclination. It is clear that the total quantity of PPs in the extracted solution mainly depends on EtOH and the extraction temperature.

### 3.4. Effect of Solvent Concentration

The concentration of the solvent in the extraction solution plays a significant role. A solvent is a reactor in extraction, while temperature and time of extraction are the conditions. Thus, it can be said that the final response mainly depends on the concentration of the solvent. The main effect plots show that NaOH and EtOH solvents increment increase the quantity of polyphenols in the extracted solutions up to a specific point. In the case of NaOH solvent, the quantity of PPs in the extraction solutions increased by increasing the NaOH concentration in water. The trend went up to 9 g/L concentration with a maximum yield of PPs, i.e., 19.48%. Further increase in concentration to 12 g/L caused the yield to reduce to 16.034%. This reduction in color strength can be attributed to the excessive formation of acidic hydroxylated structure and high reactivity of coloring components in a strongly alkaline medium [33]. An increase in EtOH percentage from 0 to 75 showed a positive effect on the extracted quantity of PPs from 14.162% to 21.332%. A further increase from 75% to 100% decreased the quantity of PPs to 16.908%. Thus, we can say that 75% aqueous EtOH is optimal for extracting PPs from aerial parts of AWW. Spigno et al. reported the decrease in total phenols extracted from grape marc [34], and Prasad et al. noticed the behavior in their study of extraction from longan fruit pericarp [35].

### 3.5. Effect of Temperature

The optimal extraction temperature in the present study for both solvents was 85 °C, with a PP yield of 19.42% for NaOH/water and 20.04% for EtOH/water solvent systems. Increasing the temperature beyond 85 °C caused a decrease in PP yield from 19.42 to 16.88 for NaOH/water and from 20.04 to 17.84 for EtOH/water. An initial increase up to the optimum temperature is due to the disruption of plant tissues’ cell walls, which makes the dissolution of solutes easy [36], but a further increase in temperature beyond the optimum limit possibly degrades the phytochemicals, as natural compounds are temperature sensitive.

### 3.6. Effect of Time

Several studies have shown that the extraction yield of PPs depends on the nature of the plant material subjected to extraction, the solvent type and concentration used, and the temperature at which extraction took place. Maximum PP yield from different materials has been achieved at different time durations, i.e., 10 min for peanut seed extraction and 180 min for dried sage extraction [37]. During our study, the PP yield in the extraction solutions increased from 14.89% to 18.16% when extraction time was prolonged from 20 min to 80 min using NaOH as the solvent. A further increase in time to 100 min decreased the yield from 18.16% to 17.87%. Using EtOH as a solvent, the optimum time of extraction was 100 min, with a maximum PP yield of 18.74%. The yield was 14.236% at 20 min. More prolonged exposure of the extraction material to air causes the oxidation of PPs, resulting in reduced output.

### 3.7. Analysis of Variance

Analysis of variance (ANOVA) determines the significance of each process parameter taken for the optimization according to the obtained response. Significance depends on two values, namely *p*-value and F-value. If the *p*-value is less than 0.05, the parameter is considered to affect response significantly. The greater the F-value is, the greater the significance [38]. ANOVA for NaOH/Water and EtOH/Water systems is given in Table 5. The results show that extraction temperature is the most significant parameter with a *p*-value of 0.001 and an F-value of 10.78, followed by the NaOH concentration, which has a *p*-value of 0.001 and an F-value of 9.96. Extraction time in the NaOH/Water solvent system is the least significant, as it has the lowest *p*-value of 0.005 among all three parameters. However, the point to be noted is that all three parameters have *p*-values less than 0.005, making the parameters significant.

ANOVA results for the EtOH/Water solvent system are shown in Table 6. EtOH concentration and extraction temperature significantly impact the response with *p*-values 0.005 and 0.014, respectively. Extraction time is not substantial as the respective *p*-value is greater than 0.05.

### 3.8. Study the Interaction Plots

**Figure 4:**
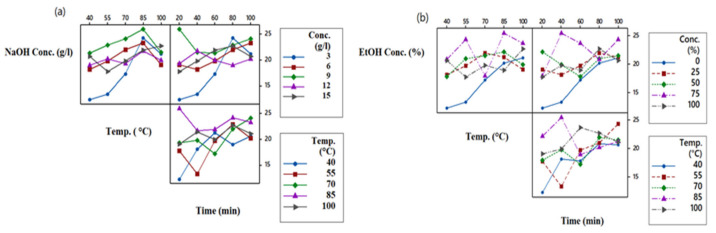
Interaction plot for (**a**) NaOH and (**b**) EtOH.

For optimization, proper selection of the initial parameters is essential. Parameters should be such that they have good interaction with each other. If there is no interaction between the selected parameters, then the whole optimization process and obtained results lose credibility. Figure 4a,b show the interaction plots between three extraction parameters such as solvent concentration, temperature, and time. All three chosen parameters are plotted against each other. The absence of parallel lines and the presence of gaps between the lines of different levels is a measure of the significance of the interaction [39]. Figure 4a shows the interaction plot between the three factors for NaOH as an extraction solvent. The most significant interaction is between temperature and time of extraction, followed by the interaction between concentration and time and the most negligible interaction between concentration and extraction temperature. The trend is as follows:

**Temp. °C − Time > Conc. (%) − Time (min) > Conc. (%) − Temp. °C**

Figure 4b represents the parameter interactions for EtOH as a solvent. It can be seen from the plots that the most significant interaction is between the concentration of EtOH and extraction time. The interaction between concentration and temperature is of the least significance. The trend of interaction is as follows:

**Conc. (%) − Time (min) > Temp. °C − Time > Conc. (%) − Temp. °C**

### 3.9. Characterization of the Dye

**Figure 5:**
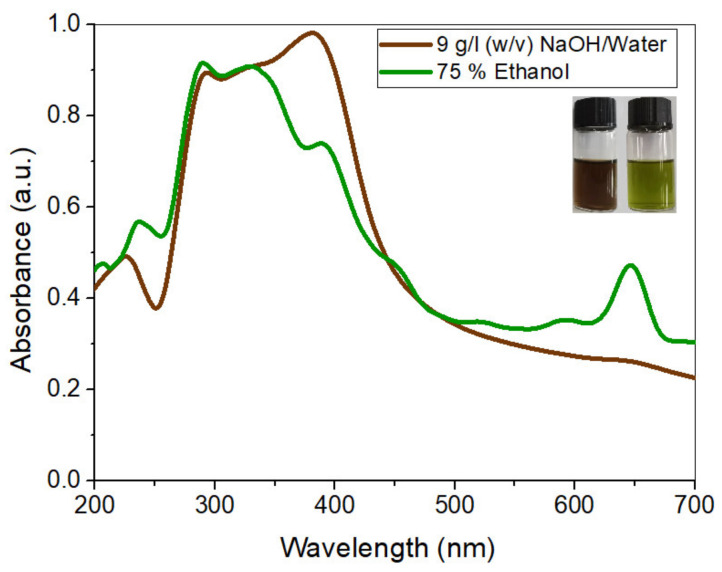
UV-Vis spectrum of optimized extracts.

The optimized extracted solutions were characterized by using UV-Visible spectrophotometry. The solutions were diluted 100 times to measure the absorbance peak values. The UV-Vis spectrum of both the solutions from 200 to 800 nm wavelengths is shown in Figure 5.

The spectrum shows that extracted dyes are mixtures of different compounds, including phytochemicals such as tannins, phenols, flavonoids, and chlorophyll. The peaks within the range of 200–300 nm show condensed tannin compounds [40], along with catechin gallates and naringenin at around 277–278 nm and 278 nm, respectively [41]. The peak at 320 nm and onward are the signature peaks of flavonoid derivatives such as apigenin, caffeic acid, jaceosidin, and eupatilin [42]. Eupatilin is the principal chemical component present in the leaves of AWW [43]. Furthermore, the smaller peaks in the region from 430 to 450 nm and the higher peak in area 640–660 nm in the EtOH extract’s spectrum show chlorophyll [44]. It exerts a greenish shade [45] in the dyed cotton fabric samples.

**Figure 6:**
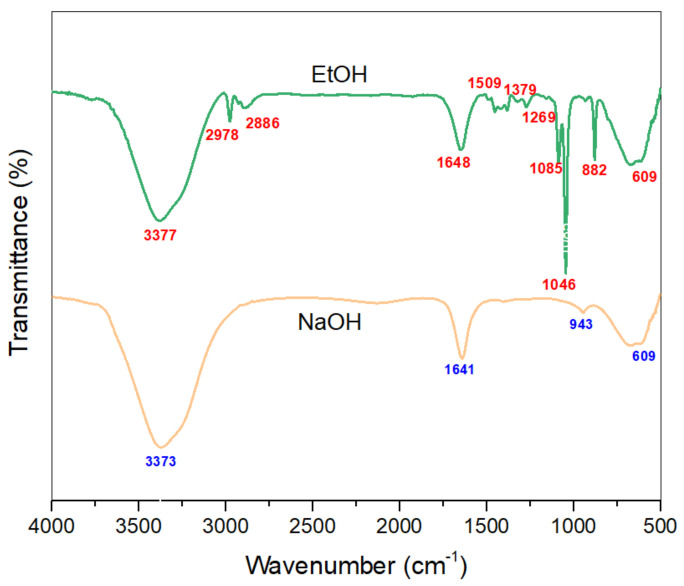
FTIR spectra of the optimized extracts.

The FTIR spectra of the optimized extracts of NaOH/Water and EtOH/Water are given in Figure 6. The wide peak within the area 3550–3100 cm^−1^ is characteristic of the –OH stretching vibration of benzene rings and methylol groups of phenolic structures like tannins and flavonoids [46]. The peaks around 2960 and 2890 cm^−1^ are due to symmetrical and asymmetrical vibrations of -CH_3_ groups. Small peaks around 1260–1085 cm^−1^ show the presence of aromatic -C-C- and –C-O- stretch of phenols. This placement shows that the –OCH_3_ group is present on the aromatic system which refers to eupatilin and jaceosidin [47].

In addition, the peak at 1648 cm^−1^ refers to –C=C- stretching vibration, which conjugates with the –C=O- stretch of flavonoid and tannin groups. The peak at 1453 cm^−1^ reflects the –C-H- stretching with –OH deformation. The peak at 1370 cm^−1^ is because of the –C=O- stretching of the phenolic groups. The peak at 1509 cm^−1^ (-C-C- aromatic stretch), along with multiple peaks around 950–670 cm^−1^ (aromatic –C-H- bending), is due to the presence of an aromatic system [48]. Moreover, the peak at 609 cm^−1^ can be attributed to the bending vibrations of aromatic compounds.

FTIR spectra along with UV-Vis spectrophotometry results are in accordance with the literature, and thus strengthen the claim that the eupatilin and chlorophyll are present in the extracts and imparted colors to the dyed cotton fabrics.

**Figure 7:**
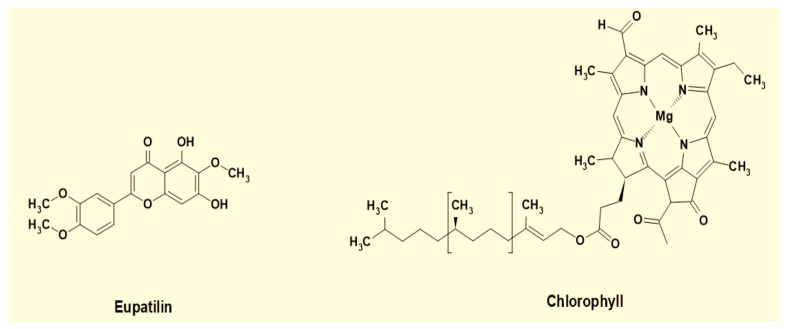
Molecular structure of major components in the extracts.

The molecular structures of eupatilin and chlorophyll are given in Figure 7.

### 3.10. Optimization of Dyeing Conditions

**Figure 8:**
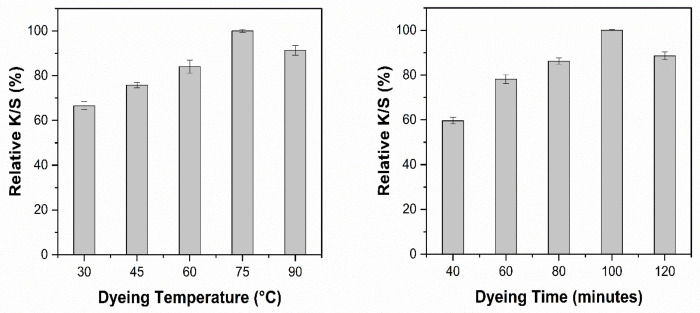
Effect of dyeing conditions on the relative K/S obtained with NaOH extract.

**Figure 9:**
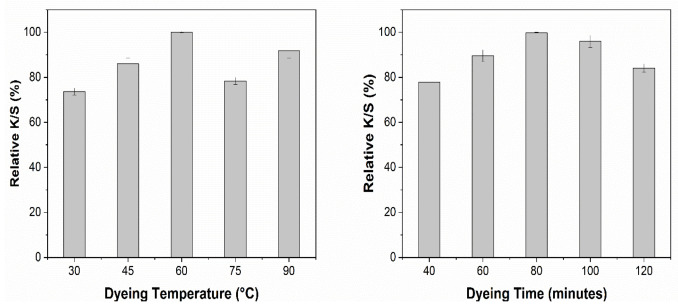
Effect of dyeing conditions on the relative K/S obtained with EtOH extract.

Cotton fabric was cationized by using 30 g/L CHPTAC agent before dyeing. Dyeing conditions were optimized based on color strength (K/S). All the samples were dyed with optimized extracts with a material to liquor ratio of 1:20. The pH of the extracts was kept unchanged. The effect of different dyeing temperatures and dyeing time on the K/S obtained using NaOH and EtOH solvent systems is shown in Figure 8 and Figure 9, respectively.

It is evident that increasing the dyeing temperature from 30 °C to 75 °C had a positive effect on the K/S (Figure 8). Further increase in the dyeing temperature decreased the color strength by 9%. Figure 8 shows that the dyeing time almost had the same effect as the dyeing temperature. An increase in the K/S was observed up to 100 min, but a further increase in the dyeing temperature reduced the color strength (K/S). For dyeing of cationized cotton fabric with 9 g/L NaOH extract, the optimum color strength can be achieved by dyeing at 75 °C for 100 min.

Figure 9 shows the effect of dyeing temperature and dyeing time for the cotton fabric treated with the EtOH solvent extract, which had the same effect as that of the NaOH solvent extract but for ETOH solvent extracts, the maximum K/S obtained at lower temperature and shorter time compared to the NaOH solvent extracts. The optimized dyeing conditions for the ethanol solvent system are 60 °C and 80 min, respectively. The increase in K/S by increasing temperature up to a certain level can be attributed to the better exhaustion of coloring molecules. However, an increase beyond the suitable temperature caused the decrease in color strength and non-uniformity in obtained color. This effect can be attributed to the hydrolytic degradation of dye molecules at high temperatures [49].

Moreover, dyeing time plays a great role in the equilibrium absorption of dye molecules in the dyebath and fiber surface. As we put the fabric into the solution, the molecules start moving from solution to fiber. This process keeps going on until the equilibrium is achieved. Further increase in the dyeing time causes desorption of the dye molecules already absorbed on to fiber surface and hence causes a decrease in K/S and uniformity of the color [50].

### 3.11. Effect of Mordanting

Pre-mordanting of cationized cotton fabric was performed by using salts of copper and iron metals and the effects on the color coordinates L*, a*, b*, C, and h and color strength K/S of the dyed fabrics with the optimized solution at optimized dyeing conditions are given in Table 7. The color strength increased for both the dye solution obtained through alkaline extraction and ethanolic extraction. The highest K/S value of 14.36 was obtained for the fabric treated with the EtOH extract by using iron as a mordant, which can be attributed to the stable chelate formation tendency of d-block elements [51]. A decrease in purity of color (Chroma C*) was observed after mordanting with iron [52]. For NaOH extract, a slight increase in lightness was observed when copper was used as a mordant. In all other cases, lightness is decreased. Mordanting increases the adsorption of dye on the fabric surface [53] and thus can be attributed as the reason for a decrease in lightness. Furthermore, there is the slightest change in hue of color for NaOH extract, but a notable difference in the shade was observed when mordants were used for dyeing with EtOH extract. It is clear from Table 7 that the color shades obtained with and without mordanting are different. This difference can be attributed to the dye–metal ion chelation system, which is not available in non-mordanted fabric.

### 3.12. Fastness Properties

Colorfastness to rubbing, washing and light of the cotton fabrics dyed with the extracted phytochemicals with and without mordanting agents are presented in Table 8. For both NaOH and EtOH extracted dyes, the rubbing and wash fastness properties remain the same even after using copper and iron as mordants through pre-mordanting. This can be attributed to the presence of tannin compounds in the dye solution, which also act as a natural mordant [54]. Light fastness properties of the NaOH extract dyed sample pre-mordanted with iron showed a slight decrease. The reason could be the catalytic effect of iron on coloring components [55]. For EtOH extracted dye, the light fastness properties are relatively low as green colored chlorophyll is destroyed easily [2]. After pre-mordanting, the light fastness values were improved on the blue wool scale from “1–2” to “3” by using copper and to “4” by using iron as a mordant. The effect can be attributed to the formation of metal ion coordinated complexes, making strong chelation with dye molecules [56].

### 3.13. Functional Finishing Properties

#### 3.13.1. Antibacterial Activity

**Figure 10:**
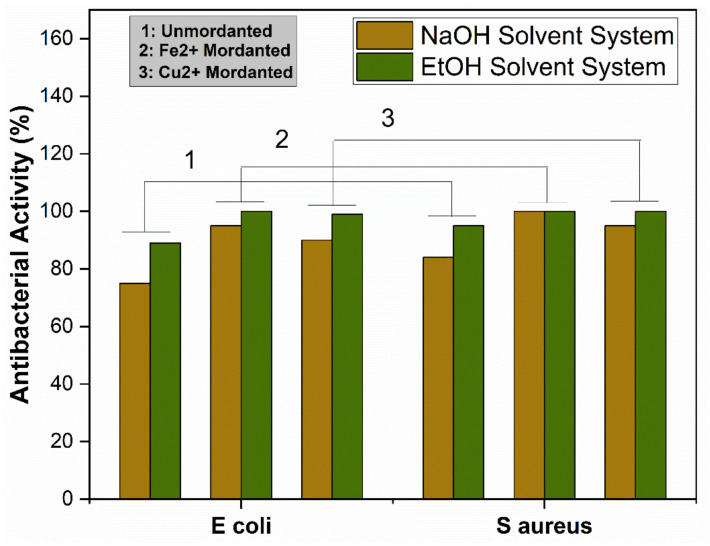
Antibacterial activity of unmordanted and mordanted dyed fabrics.

The antibacterial activity of the extracted dye solutions was tested through the turbidity method. Fabric samples were dyed with and without using the metallic mordants and examined. The ability of the extracted dyes was measured against two different kinds of bacteria, namely *E. coli* and *S. aureus*. Antibacterial activities of dyed fabric and pre-mordanted dyed fabrics are shown in Figure 10.

**Figure 11:**
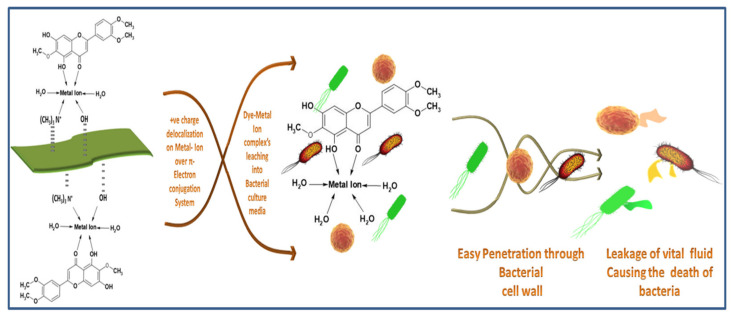
Antibacterial mechanism of the Dye–Metal complex.

Extracted dyes showed good antibacterial activity, which was higher against *S. aureus* than *E. coli*. The reason is that *E. coli* is a Gram-negative bacterium, which has an extra protective layer of phospholipid containing negatively charged lipopolysaccharide, protecting the Gram-negative bacteria against polyphenols [57]. The exact antibacterial mechanism of the natural dyes is unknown [58]. However, some studies have shown that hydrogen bonding and hydrophobic reactions between polyphenols and proteins of bacteria damage the bacterial cell wall, causing the leakage of cell fluids, and ultimately killing the bacteria. Chelation of metal ions with dye molecules decreases the polarity of metal ions because of the sharing of positive charges throughout the dye–ion system. This sharing of positive control makes the π electron highly delocalized over the chelating ring, increasing the lipophilic nature of the central metal atom, making it more permeable into the bacterial lipid layer [59]. According to Overtone’s concept [60] and the Tweedy chelation theory [61], only lipid-soluble materials can pass through the lipid protective layer of bacteria. The proposed mechanism of antibacterial activity of the dyed fabric is given in Figure 11.

#### 3.13.2. UV Protection

Textiles are frequently used to provide UV protection because of their inherent light resistance. Additionally, due to the high surface area of textile fabrics when compared to other materials, they are more vulnerable to damage from ultraviolet radiation. Generally, there are three ultraviolet radiation ranges, including the UV-A band (320–400 nm); the UV-B band (290–320 nm); and the UV-C band (200–290 nm). UV-A and UV-B are mostly responsible for UV protection phenomenon and determined with UPF (ultraviolet protection factor) rating.

The UPF rating and percentage transmission values (T%) of UV-A and UV-B radiations through the cotton fabric dyed with the extracted colorant solutions (aqueous ethanolic and NaOH/Water solutions) are given in Table 9. It can be seen that the fabric dyed with ethanolic dye solution achieved better results than the fabrics dyed with the NaOH dye system. The difference in the protection level can be attributed to the difference in the metal ion–dye–fabric chelation system and the difference in phytochemical components extracted in both solutions [62].

## 4. Conclusions

Polyphenols were extracted from the aerial parts of AWW by an aqueous extraction technique using NaOH/Water and EtOH/Water solvent systems. Extraction using 9 g/L NaOH at 85 °C for 20 min and 75% EtOH at 85 °C for 40 min was optimal for NaOH and EtOH solvent systems, respectively. The polyphenols extracted by the NaOH/water extraction method produced brown color and the EtOH/water extraction method produced green color shade on the cotton fabric. The treated fabric exhibited very good antibacterial and UV protective properties. The pre-mordanting of the fabric with iron and copper salt increased the colorfastness to light and washing of the dyed cotton fabrics and also further enhanced antibacterial activity. The color strength obtained with aqueous EtOH extract is higher than the NaOH/Water system as EtOH extract contains a high quantity of polyphenols. A maximum K/S value of 14 was obtained with iron pre-mordanting for EtOH/Water extracted dye. The current study has proved that the hectic and time-consuming extraction of colorants from natural resources can be optimized by Taguchi experimental design without doing countless hit and trial experiments.

It is possible to achieve different colors by using different solvent extraction system and also by using different mordanting agents. Thus, it can be concluded from the current study that AWW medicinal plant is a good source of functional bio-colorant for cotton fabric.

## Figures and Tables

**Table 1:** Experimental parameters for trials.

Type of Solvent	Solvents (mL)	Temperature (°C)	Time (min)
Water	100	100	60
Water/HCl	90/10	80	60
Water/NaOH	90/10	80	60
EtOH	100	70	60

**Table 2:** Color coordinates of extracted solutions.

Extraction System	K/S	L*	a*	b*	C*	h°
Water	2.3131	17.03	3.74	8.64	9.41	66.56
Acidic Water	2.3694	15.27	13.85	19.94	24.28	55.20
Alkaline Water	3.7219	11.98	0.59	8.92	8.94	86.23
Aqueous Ethanol	3.0975	6.36	−1.80	7.40	7.61	103.64

K/S is the strength of the color on the fabric, L* represents the lightness, a* represents the redness (positive) or greenness (negative), b* represents yellowness (positive) or blueness (negative), C and h° stand for chroma and hue of the color, respectively.

**Table 3:** Extraction parameters and their levels.

Parameters	Units	Levels				
		Level 1	Level 2	Level 3	Level 4	Level 5
NaOH Conc.	g/L	3	6	9	12	15
EtOH Conc.	%	0	25	50	75	100
Temperature	°C	40	55	70	85	100
Time	Min	30	45	60	75	90

**Table 4:** Taguchi DOE with Factors and obtained response for NaOH and EtOH solvent systems.

Exp. Number	Factors				Polyphenols Components	
	Solvent Concentration		Temperature	Time	NaOH	EtOH
	NaOH	EtOH				
	g/L	%	°C	min	%	%
1	3	0	40	20	8.31	8.36
2	3	0	55	40	9.38	10.94
3	3	0	70	60	13.27	14.59
4	3	0	85	80	20.22	19.56
5	3	0	100	100	17.13	17.36
6	6	25	40	40	14.14	13.87
7	6	25	55	60	15.75	16.99
8	6	25	70	80	18.02	17.52
9	6	25	85	100	19.27	20.2
10	6	25	100	20	15.08	15.98
11	9	50	40	60	17.31	12.98
12	9	50	55	80	18.86	17.9
13	9	50	70	100	20.13	19.21
14	9	50	85	20	21.96	15.02
15	9	50	100	40	17.48	18.1
16	12	75	40	80	14.98	18.91
17	12	75	55	100	16.19	22.39
18	12	75	70	20	15.32	16.05
19	12	75	85	40	17.7	25.53
20	12	75	100	60	15.98	23.78
21	15	100	40	100	16.64	14.56
22	15	100	55	20	13.76	15.77
23	15	100	70	40	15.8	20.33
24	15	100	85	60	17.94	19.91
25	15	100	100	80	18.74	13.97

**Table 5:** ANOVA for NaOH solvent system parameters.

Source	Degree of Freedom	Adjacent SS	Adjacent MS	F-Value	*P*-Value
NaOH Conc. (g/L)	4	76.06	19.016	9.96	0.001
Temp. (°C)	4	82.29	20.574	10.78	0.001
Time(min)	4	49.70	12.425	6.51	0.005
Error	12	22.91	1.909		
Total	24	230.97			

**Table 6:** ANOVA for EtOH solvent system.

Source	Degree of Freedom	Adjacent SS	Adjacent MS	F-Value	*P*-Value
EtOH Conc. (%)	4	133.91	33.478	6.38	0.005
Temp. (°C)	4	103.86	25.964	4.95	0.014
Time(min)	4	59.08	14.771	2.81	0.074
Error	12	62.97	5.247		
Total	24	359.82			

**Table 7:** Color properties of dyed cationized cotton fabric with and without pre-mordanting.

Solvent	Mordant	Shade	K/S	L*	a*	b*	C*	h
NaOH	Without mordant	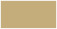	2.3812	69.15	4.27	26.25	26.59	80.77
	FeSO_4_	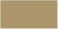	4.3288	61.45	5.59	23.56	24.21	76.64
	CuSO_4_	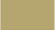	3.3037	66.18	0.36	27.79	27.80	89.26
EtOH	Without mordant	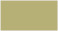	2.9451	68.68	−3.02	27.80	27.96	96.19
	FeSO_4_	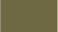	14.3600	44.39	−1.37	20.15	20.20	93.88
	CuSO_4_	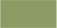	5.0319	59.99	−12.05	24.55	27.35	116.15

K/S is the strength of the color on the fabric, L* represents the lightness, a* represents the redness (positive) or greenness (negative), b* represents yellowness (positive) or blueness (negative), C and ho stand for chroma and hue of the color, respectively.

**Table 8:** Colorfastness properties of dyed cationized cotton fabric with and without pre-mordanting.

Solvent	Mordant	Colorfastness to Rubbing		Colorfastness to Washing		Light Fastness
		Dry	Wet	Color Change	Cotton Staining	
NaOH	Without mordant	4–5	4	4	4	4–5
	CuSO_4_	4–5	4	4–5	5	4–5
	FeSO_4_	4–5	3–4	4–5	4	3–4
Ethanol	Without mordant	5	3–4	3	4	1–2
	CuSO_4_	4–5	4	4	4	3
	FeSO_4_	4–5	4–5	4	4–5	3–4

**Table 9:** UV protection of unmordanted and mordanted dyed fabrics.

		Transmittance (T%)		UPF
		UV-A	UV-B	
NaOH/Water	Unmordanted	438.22	15.48	45
	Cu^2+^	154.03	1.16	63
	Fe^2+^	43.36	0.05	617
EtOH/Water	Unmordanted	4.35	0.08	630
	Cu^2+^	0.58	0.05	1253
	Fe^2+^	0.05	0.05	2000

## Data Availability

Data will be provided by the corresponding authors upon request.
